# Evidence of a hierarchical representation in bodily self-consciousness: the neural correlates of embodiment and presence in virtual worlds

**DOI:** 10.3389/fnhum.2025.1468947

**Published:** 2025-04-01

**Authors:** Evan Alexander Owens, Robert O. Duncan

**Affiliations:** ^1^Behavioral and Cognitive Neuroscience, Department of Psychology, The Graduate Center, The City University of New York, New York, NY, United States; ^2^Cognitive Neuroscience Laboratory, Department of Behavioral Sciences, York College, The City University of New York, Jamaica, NY, United States

**Keywords:** bodily self-consciousness, embodiment, presence, multisensory integration, virtual reality, functional magnetic resonance imaging (fMRI)

## Abstract

**Introduction:**

Bodily Self-Consciousness (BSC) is the perception of bodily awareness that arises from the integration of neuronal signals in multiple sensory modalities. BSC is composed of embodiment (the perception of owning a body) and presence (the perception of being at a location). Converging lines of evidence suggest embodiment and presence are supported by different neural networks. Several models have been proposed to describe how BSC manifests from these networks, but how these networks interact is not fully understood. We propose that the perception of presence is predicated upon the perception of embodiment. Specifically, even though neural networks for presence and embodiment partially overlap, there exists a subset of brain areas that mediate the flow of information from those supporting embodiment to those supporting presence.

**Methods:**

To test this model, sensory feedback was manipulated in a virtual environment to affect BSC, while measuring behavioral performance and physiological responses in relevant brain areas. Correlated versus uncorrelated feedback was used to manipulate perceptions of embodiment. First- versus third-person perspective was used to manipulate perceptions of presence.

**Results:**

Mean reaction times and accuracy were better with correlated feedback and first-person perspective. Functional magnetic resonance imaging (fMRI) measurements of neuronal activity identified frontoparietal and temporoparietal brain areas that appear to support embodiment and presence, respectively. We compared the effect of embodiment manipulations on presence areas and vice versa. The effect sizes for manipulations of embodiment were greater than those for manipulations of presence. This trend was also observed for brain areas that appeared to encode both embodiment and presence.

**Discussion:**

This data indicates that networks associated with embodiment and presence overlap, and brain areas that support presence may depend upon the activity of those that support embodiment.

## 1 Introduction

Sensations arising from multiple modalities are filtered by neural mechanisms for attention to create perceptions that enter and, indeed, define the conscious mind (Damasio, 1999; Riva et al., 2015). The integration of visual, auditory, tactile, vestibular, and proprioceptive signals manifests in an awareness of the body that is referred to as bodily self-consciousness (BSC). This self-awareness enables us to identify where we are in space and time, which is necessary to execute goal-oriented behaviors. BSC is thought to derive from the subjective experiences of *embodiment* and *presence* (Blanke et al., 2015). Embodiment is the feeling of ownership toward a real or virtual extremity (Arzy et al., 2006; Blanke, 2012). Whereas presence is the perception of being physically located in a real or virtual space (Ehrsson, 2007; Ionta et al., 2011; Petkova and Ehrsson, 2008; Slater et al., 2010).

Behavioral studies indicate that changes to perceived embodiment may derive from a conflict between different sensory modalities (Armel and Ramachandran, 2003; Botvinick and Cohen, 1998; Costantini and Haggard, 2007; Liepelt et al., 2017; Lloyd, 2007; Kalckert and Ehrsson, 2017; Ramachandran et al., 1995; Weser et al., 2017). Early studies of embodiment explored sensory conflict using the rubber hand illusion (Armel and Ramachandran, 2003; Botvinick and Cohen, 1998). In this illusion, a rubber hand is placed near one of the participant’s hands, which is occluded from view. Synchronous stroking of both the real, hidden hand and the rubber hand causes participants to feel as if the rubber hand is their own. Similar illusions of embodiment have also been used to alter perception of the face and full body (Apps and Tsakiris, 2014; Blanke, 2012; Ehrsson, 2012; Makin et al., 2007). These embodiment illusions have since been adapted for neuroimaging (Costantini and Haggard, 2007; Kalckert and Ehrsson, 2017; Liepelt et al., 2017; Lloyd, 2007; Weser et al., 2017), and other technologies such as virtual and augmented reality have become useful tools in the exploration of this subjective phenomenon (Perez-Marcos et al., 2012; Slater et al., 2008, 2009, 2010). For example, Kilteni et al. (2012) revealed that congruent sensorimotor feedback can distort the perception of embodiment for virtual extremities presented in virtual reality. Specifically, when a person wearing a head-mounted display (HMD) is presented with a real-time rendering of a virtual hand or arm in the same location as their actual arm, the participant can embody a virtual arm three times as long as their own. Like the rubber hand illusion, attention and multisensory conflict are thought to be at the root of this effect.

Multicellular recording and neuroimaging studies suggest multiple neural networks contribute to the perception of embodiment, and it is believed that embodiment is primarily associated with frontoparietal networks in the cortex (Graziano et al., 1999; Guterstam et al., 2015; Guterstam et al., 2019; Mountcastle et al., 1975; Petkova et al., 2011a; Rizzolatti et al., 1997). Multimodal neurons in the parietal lobe, somatosensory cortex, and precentral cortex were activated when the monkeys viewed either a real or fake arm, and these neurons also became active when these arms were touched (Graziano et al., 2000, 2002). Neuroimaging studies of limb ownership suggest that the ventral premotor cortex and intraparietal sulcus are primary contributors to the overall experience of embodiment (Blanke et al., 2015; Guterstam et al., 2015, 2019). Areas that are also correlated with embodiment include the insula, precuneus, post central sulcus, parietal lobe, and posterior cingulate cortex (Ehrsson et al., 2004, 2007; Farrer et al., 2003; Gentile et al., 2013; Guterstam et al., 2015; Kanayama et al., 2009; Limanowski and Blankenburg, 2016).

Virtual reality with HMDs is also an effective tool for altering the subjective experience of presence. In these studies, participants might view their own body from behind (i.e., third-person perspective), or they might view a scene from the first-person perspective of a mannequin or virtual body (e.g., Henrique et al., 2017; Petkova and Ehrsson, 2008; Petkova et al., 2011b; Maselli and Slater, 2013). Using such paradigms, participants perceived that their body was at a virtual location in the scene (e.g., Huang et al., 2017; Lenggenhager et al., 2007, 2009). These studies suggest that presence is the product of multisensory conflict, in which the perceived tactile stimulation and visual cues are sufficient to induce experiences of drift or relocation.

Electrophysiological and neuroimaging studies in humans have provided evidence that temporoparietal regions in the cortex are involved in the perception of presence (Arzy et al., 2006; Blanke et al., 2015; Ionta et al., 2011, 2014; Slater et al., 2010). EEG and fMRI studies have demonstrated that altered perceptions of presence are associated with the activity in the medial and dorsal prefrontal cortices, the parietal lobe, and the precuneus (Baumgartner et al., 2006, 2008; Lenggenhager et al., 2011; Sharma et al., 2017). In an fMRI study, Ionta et al. (2011) had participants perform a mental ball-dropping task from various third-person perspectives. Data from this study suggest that changes in perceived self-location were correlated with activity in the temporoparietal junction. Compared to asynchronous back-stroking conditions, synchronous conditions from this study were correlated with greater activity in temporoparietal junction. Other areas that also correspond with distortions in presence include the extrastriate body area, insular cortex, hippocampus, parietal lobe, and superior temporal gyrus (Arzy et al., 2006; Blanke et al., 2015; Corradi-Dell’Acqua et al., 2008; Ionta et al., 2011, 2014; Guterstam et al., 2015).

Several models have been proposed to explain BSC and its components, embodiment and presence. Theories describing BSC can be divided into coupled and decoupled network theories. Coupled network theories propose embodiment and presence share neural networks, at least in part (Blanke et al., 2015; Guterstam et al., 2015; Park and Blanke, 2019). Decoupled network theories propose that embodiment and presence do not share overlapping neural networks (Longo et al., 2008; Maselli and Slater, 2014; Serino et al., 2013). Some coupled network models have proposed that posterior cingulate cortex mediates activity between brain regions associated with embodiment and presence (Guterstam et al., 2015), whereas others have argued intraparietal sulcus and insula are better candidates for this role (Park and Blanke, 2019). Recent evidence also suggests the anterior precuneus might be critical (Lyu et al., 2023).

The coupled model proposed by Guterstam et al. (2015) is supported by evidence from their fMRI study, in which they manipulated both presence and embodiment. To alter experiences of embodiment, congruent or incongruent vibrotactile stimulation was administered to the body. To alter the perception of presence, the position of the camera feeding into the participant’s HMD was moved about the MRI scanner room. Data indicate that changes to perceptions of embodiment were correlated with changes to activity in the ventral premotor cortex, lateral occipital cortex, and intraparietal sulcus. On the other hand, changes to perceptions of presence co-varied with changes to activity in the hippocampus, intraparietal sulcus, precuneus, and retrosplenial cortex. An effective connectivity analysis revealed that the posterior cingulate cortex and intraparietal sulcus were highly correlated with both presence and embodiment conditions. The authors concluded that posterior cingulate cortex likely governs the interplay between networks supporting embodiment and presence. Park and Blanke (2019) also support a coupled network model. However, they assert that the temporoparietal junction is a greater contributor to the perception of presence, while the interparietal sulcus and insular cortex link subjective experiences of embodiment and presence.

Decoupled network models propose that perceptions of embodiment and presence do not share overlapping neural networks. Serino et al. (2013) argued that embodiment and presence are supported by different neural mechanisms. This argument is based on results from neuroimaging studies and clinical studies of somatoparaphrenia and autoscopic phenomena, two pathological dissociative perceptions. First, Petkova et al. (2011a) found body ownership was correlated with activity in ventral premotor and posterior parietal cortex. Second, Ionta et al. (2011) found self-location to be correlated with activity in temporoparietal junction but not in premotor or posterior parietal cortex. Consequently, Serino et al. (2013) asserted this double dissociation supports a decoupled model of BSC. Further support of this model comes from clinical studies that have associated dysfunction in perceived self-location with lesions in the temporoparietal junction (Blanke et al., 2002). And, in the case of heautoscopy, patients feel disturbances in perceived self-location even when their sense of embodiment is intact (Heydrich and Blanke, 2013). Finally, Maselli and Slater (2014) found support for decoupled models by manipulating visuo-tactile and visual sensorimotor cues in virtual reality. Perceptions of self-location and body ownership could be independently altered, supporting the assumption that embodiment and presence have dissociated networks. However, their findings did suggest the enhancements to the perception of embodiment may “boost” perceived presence. So, they admit it is possible that these networks could coexist in parallel.

Taking these models into account, we investigated the physiological correlates of embodiment and presence in virtual environments. It was predicted that alterations to embodiment would correlate with activity in the ventral premotor cortex, intraparietal sulcus, and lateral occipital cortex. Distortions of perceived presence were predicted to correlate with activity in the temporoparietal junction, supramarginal gyrus, and the hippocampus. Activity in multi-modal brain areas like the insula, posterior cingulate cortex, the intraparietal sulcus, and possibly the extrastriate body area were predicted to correlate with both presence and embodiment. The bilateral insular cortex, the left and right posterior cingulate cortex, intraparietal sulcus, and the extrastriate body area have been implicated in the culmination of embodiment and presence in many of the studies mentioned (e.g., Blanke et al., 2015; Guterstam et al., 2015; Park and Blanke, 2019). The insula is composed of multimodal neurons that encode vestibular activation, proprioception, vision, and touch, and is linked to many regions in the frontal, parietal, and temporal lobes. The posterior cingulate cortex has been associated with a multitude of functions related to BSC, including attention and memory. Multisensory neurons in the intraparietal sulcus respond to visuo-tactile stimuli near or approaching specific body parts, including the hand and face. The extrastriate body area is linked to the perception of a human body, and it has been shown to be significantly influenced during changes in perceived embodiment and presence. Unlike previous coupled models, it is predicted that the perception of presence is predicated upon the perception of embodiment. We posit that one cannot perceive to be *somewhere* unless they perceive to be *something*. Therefore, manipulations that alter neural networks supporting embodiment may affect downstream networks encoding presence, but the reverse is less likely to be true.

## 2 Materials and methods

### 2.1 Psychophysical experiments

We developed a novel paradigm to investigate the neural correlates of BSC in the MRI environment, where participants are typically not allowed to move. It was predicted that relatively low-embodiment conditions would result in decreased performance compared to high-embodiment conditions. It was also predicted that relatively low-presence conditions would result in decreased performance in the task compared to high-presence conditions.

#### 2.1.1 Participants

This study was a within-subjects design. Participants were right-handed men (*n* = 5) and women (*n* = 6) between the ages of 27 and 57 years old (Mean = 33.81 years old). Additional demographic data was not collected. Participants were not excluded for sex, race, or gender. Participants were excluded if they were left-handed. They were compensated $60 for their participation. Informed and written consent was obtained after the nature and possible consequences of the study were explained. All policies and procedures complied with the APA ethical standards, the World Medical Association’s Declaration of Helsinki, and were approved by the York College Internal Review Board.

#### 2.1.2 Apparatus

Stimulus creation and data management were conducted using a laptop computer [MSI Intel(R) Core i7 2.8 Hz; 32Gb RAM; Windows 10 Pro operating system] and the Unity game development engine (version 2020.1.0f1) (Unity Technologies, San Francisco). Visual stimuli were presented using a Vive Pro HMD (HTC Corporation, Bellevue, Washington), which provide users with a fully immersive experience (1,440 × 1,600 pixels per eye, 90 Hz refresh rate, 110° FOV, < 10 ms latency). Hand position was monitored using a Vive Tracker. The Vive system tracks position relative to two infrared light-emitting diodes positioned equidistant from the participant. Tracked positions are used to update game objects in the virtual world with high spatial precision and low latency. Seated participants held a small Vive Tracker in their right palm, which was placed flat on the table in front of them. The Vive Tracker was also represented in the virtual environment by an identical game object that could be positioned by the virtual hand. Inverse kinematics in the game engine were used to move the avatar’s arm to any position designated by the movement of the tracker.

**FIGURE 1:**
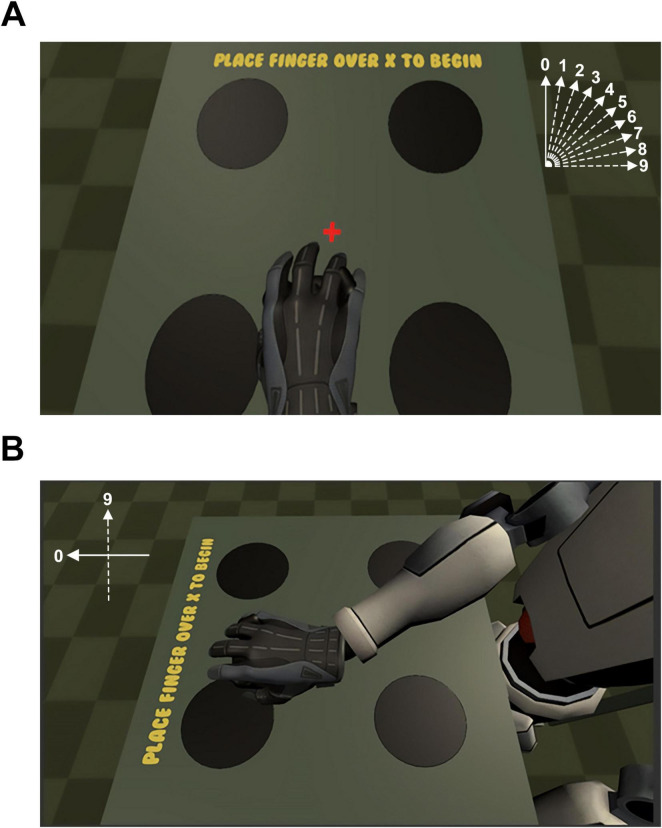
**(A)** During the high-embodiment condition, motion between the real and virtual arm were correlated, meaning that no error was introduced in the position or velocity of the virtual arm upon movement of the real arm. During the low-embodiment condition, an error term was added to the vector representing the velocity of the real arm such that the position and velocity of the virtual arm were biased according to the angle for a given step size (e.g., a step size of “4” resulted in a 40° clockwise rotation of the vector of the virtual arm). The vector representing the error for each condition is presented schematically top-right. **(B)** Step 9 of the presence conditions. For the presence conditions, the 0-step presence condition was identical to the 0-step embodiment condition. However, during the remaining presence conditions, the virtual camera was rotated between 10° and 90° according to the step size for that trial so that the participant viewed their virtual avatar from a rotated, third-person perspective. The error signal representing the error for the 90 degree rotation of the scene is presented schematically top-left.

There were two main conditions for this experiment: Embodiment and Presence. Each condition had 10 discrete steps (labeled from 0 to 9). For the embodiment condition, the tracker coordinates were rotated about the y-axis (i.e., pointing up to the sky) between 0° and 90° in 10° increments (Figure 1A). At the position with the largest step size (i.e., “step 9” = 90 degrees), the vector of the avatar’s arm was at a right angle from the veridical direction of motion made by the participant. In other words, at step 9, when the image motion was rotated 90° away from the veridical motion of the arm, the display would move the virtual arm to the “right” when the subject moved their arm “forward.” For the presence condition, the position of the camera was rotated about the y-axis between 0° and 90° in 10° increments (Figure 1B). At the position with the largest step size (i.e., “step 9” = 90 degrees of rotation), subjects viewed their avatar from the left side of the body at a distance that was equivalent to that of the unrotated scene. For both conditions, each step was presented pseudo-randomly using interleaved ascending and descending staircases until every step for that condition was run once. After every step for a condition was complete (e.g., embodiment), the participant would begin the alternate condition (e.g., presence). The cycle was repeated until the designated number of trials were completed. Embodiment and presence conditions were counterbalanced across participants.

#### 2.1.3 Procedure

The experimental task was similar to Posner’s classic cueing paradigm (Posner, 1980), but the stimulus presentation was adapted for virtual reality. The task was used to enhance immersion and monitor the attention of participants. However, the task itself was not relevant to our hypothesis. Performance metrics were used as proxies for online subjective reports of BSC for two reasons: (1) Subjective reports assess the memory of BSC rather than BSC itself, and (2) collecting subjective reports typically diminish immersion in the virtual world by directing attention to the real world.

In the virtual scene, participants were presented with a virtual avatar seated in a chair looking downward at a table from a first-person perspective. This view corresponded with the participant’s actual position in the real world. Participants were presented with a fixation cross placed at the center of the virtual table, and four disks were placed equidistant from the fixation target (Figure 1A). To begin each trial, participants moved the tracker to place their virtual hand over the fixation target. When the trial began, a red cue would signal the eventual appearance of a target. The spatial cue reliably predicted the location of the target 80% of the time. After 2 s, a green target disk would appear in one of the four possible locations at random. The participant’s goal was to indicate the correct target on the table by gliding their virtual hand over the correct target. Accuracy and reaction time were recorded for each condition. Early hand movements (i.e., before the target was presented) and late responses (i.e., greater than 5 s) resulted in the trial starting over, and no data were collected. The experiment was done in a quiet, dimly lit environment to ensure that participants fully attended to the experimental stimulus.

### 2.2 Physiological experiments

#### 2.2.1 Subjects

The participant pool for our physiological experiments was identical to that of our psychophysical experiments with the addition of one author (E.A.O.). Participants were right-handed men and women between the ages of 27 and 57 years old with normal or corrected-to-normal acuity. The mean age for participants was 34.6 years old. No additional demographic data were collected. Participants were screened using standard fMRI criteria for ferromagnetic implants, medications, or health conditions that could harm them or affect the quality of the data. Participants were not excluded for sex, race, or gender. Participants were compensated with $60 for completing the fMRI experiment. Informed consent was obtained from all patients after the nature and possible consequences of the study were explained. All methods were approved by the York College Institutional Review Board.

#### 2.2.2 Stimuli

Stimulus presentation and behavioral data acquisition were managed by Unity’s game development engine on a laptop computer (Alienware, Intel^®^ Core™ i7-9750H 2.60 GHz, 16 GB RAM). The stimulus was similar to the original psychophysical experiments, but changes were made to optimize the paradigm for use in the MR environment. The staircase procedure was no longer required. This experiment compared conditions known to reliably support or alter perceptions of embodiment and presence (i.e., step 0 vs. step 9). The trial timing was also modified to provide enough time for participants to respond, while also assuring a minimum number of trials could be completed during 20 s blocks in a standard MRI block paradigm.

There were four conditions for this experiment: high-embodiment, low-embodiment, high-presence, and low-presence. During the high-embodiment (HE) condition, the avatar’s movements and those of the participant were 100% correlated. In the low-embodiment (LE) condition, the motion vectors associated with the computer mouse were rotated 90° like the psychophysical experiments (step 9). The high-presence (HP) condition was identical to the high-embodiment condition. However, in the low-presence (LP) condition, the position of the camera was rotated 90° from its original position, just like in the psychophysical experiments (step 9). Mean accuracy and speed of the responses were recorded for each condition. Accuracy was measured by calculating the ratio of hits to total trials during each run in the scanner. Reaction time was assessed by computing the mean reaction time for all trials.

#### 2.2.3 Procedure

Participants lay in the bore of the fMRI scanner in a supine position with a desk over their lap. They held an MR-compatible trackball at the center of the board before the experiment began. Similar to our psychophysical experiments, users were presented with a first-person perspective of a virtual avatar seated in a chair that was looking downward at a table. Stimulus presentation was designed to accommodate fMRI data acquisition in a block design, where each epoch lasted 40 s. When the experiment began, conditions would alternate between high- versus low-presence or high- versus low-embodiment every 20 s for the entirety of the run. Each run lasted roughly 4 min and 20 s (6.5 epochs). Participants placed their virtual hand over a centrally positioned fixation target to start each trial. Then, participants were presented with a red cue in one of four possible locations on the table. This red cue reliably predicted the spatial location of the target 80% of the time on valid- versus invalid-cue conditions. After 2 s, a green target would appear in one of the four possible locations at random. The participant’s goal was to indicate the correct target on the table by gliding their virtual hand over the correct target. The entire MRI session took approximately 1 h.

#### 2.2.4 General MRI methodology

The fMRI images were collected using a Siemens 3-Tesla Prisma MRI Scanner equipped with a 64-channel head/neck receiver coil at the Advanced Science Research Center in New York City. Participants lay supine in the scanner bore, and their heads were secured with padding in the “birdcage” head coil. Participants viewed the visual stimuli using an angled mirror that was affixed to the head coil. Each session started with an anatomical scan using a standard T1-weighted gradient echo pulse sequence (MP-RAGE, 1 mm × 1 mm × 1 mm resolution). Anatomical scans were used as a reference volume for each participant and to align functional data across each session.

Up to eight functional scans were acquired for each participant using a low-bandwidth EPI pulse sequence lasting 260 s (TR = 1 s, TE = 30 ms, flip angle = 52°, 39 oblique slices of 3 mm thickness and 3 mm × 3 mm in-plane resolution, 1,638 × 1,638 mm FOV). The first ten temporal frames (20 s) were discarded to avoid magnetic saturation effects. The blood-oxygen level dependent (BOLD) signal was acquired for each condition using a standard block design. Complementary conditions (e.g., HE vs. LE) were contrasted during each run in alternating half-cycles of 20 s for six cycles (excluding the first half-cycle). The order of each condition was counterbalanced in a different run (e.g., LE vs. HE) and each run was repeated, yielding a total of eight runs with four repeats for each condition (e.g., HE).

An MR-compatible widescreen display (BOLDscreen 32” LCD, Cambridge Research Systems, United Kingdom; 1,920 × 1,080 pixels, 120 Hz refresh rate, 1400:1 contrast ratio, 24-bit RGB) was used to present stimuli for this experiment. Although this study was inspired by virtual reality, visual stimuli were not presented using an HMD. Because participants are not able to move their heads in fMRI, there was no need to update the visual image in response to head movement. Dichoptic presentation was also not used because immersion was presumed to be primarily driven by perception-action feedback loops rather than stereopsis. Immersion was further supported by removing all sensory distractions that could potentially compete with the stimulus. Specifically, all light was turned off in the scanner room, and curtains were placed over the monitor and at the entry to the scanner bore to prevent light from penetrating.

Responses were indicated using an MR-compatible computer mouse (MR Trackball 2, Cambridge Systems, United Kingdom) that was flipped upside-down so that it could roll smoothly across a desk placed across the participant’s lap. The trackball’s coordinates were tracked with near-zero latency and used to position the avatar’s hand in virtual space. The trackball was manipulated using the palm of the dominant right hand. The thumb and fingers were only used to grip the mouse. At the beginning of each trial, participants positioned the virtual hands at the red “x” by positioning their real hand at the center of the desk (Figure 1). The center of the desk was positioned at the participant’s waist. The sensitivity of the trackball was controlled by the computer and game engine; the effective dots per inch (eDPI) of the trackball was 800 DPI. Moving the trackball 60 mm in the real world moved the virtual hand 1,920 pixels.

#### 2.2.5 fMRI data processing

Analysis of Functional NeuroImages (AFNI) was used to prepare and analyze the fMRI data (Cox, 1996). DICOM images from the scanner were converted to AFNI BRIK files. Non-linear warping was used to align anatomical data to the standard MNI atlas (MNI152_T1_2009c) (Fonov et al., 2009, 2011). EPI data were processed using a standard protocol for block designs in AFNI. Data corresponding to the first half-cycle was removed from each run. Outlier TRs were removed from each time-series, and data were shifted to align the slice timing. Non-linear warping was used to register the functional data to the anatomical reference volume. A full-width, half-maximum (FWHM) Gaussian blur of 4 mm was applied to the data from each voxel. The data from each time series was scaled, and the mean for each run was computed. Motion parameters were computed by taking the derivative of deviations from the mean and used to censor TRs with excessive motion. The resulting uncensored EPI data were regressed using 3dDeconvolve, which performs a simple linear regression to a model generated by convolving the stimulus timing by the hemodynamic response. This output is used to compute a second voxel-wise generalized least-squares time series fit with 3dREMLfit, which results in correlation coefficients (i.e., beta weights) and t-statistics for each voxel. The units for the beta-weights of the regressor are in percent BOLD signal change.

#### 2.2.6 Regions of interest and specific hypotheses

It was predicted that the perception of presence is predicated upon the perception of embodiment. This prediction unfolds into several hypotheses. First, neural activation in several brain areas should co-vary with changes to embodiment conditions. Previous studies have shown that activity in the ventral premotor and somatosensory cortices contribute to the perception of embodiment (Gentile et al., 2015; Guterstam et al., 2015; Petkova et al., 2011a). Second, activity in a different set of brain areas should only correlate with changes to presence conditions. Studies have also suggested that the temporoparietal junction (Ionta et al., 2011; Serino et al., 2013), hippocampus (Guterstam et al., 2015), supramarginal gyrus, and precuneus (Guterstam et al., 2015) correspond with perceptions of presence. Third, brain areas that mediate the flow of information from presence and embodiment networks will respond to changes to either embodiment or presence conditions. These areas include the posterior cingulate cortex (Guterstam et al., 2015), intraparietal cortex (Ehrsson et al., 2004, 2005; Guterstam et al., 2015; Park and Blanke, 2019; Serino et al., 2013), lateral occipital cortex (Downing et al., 2001; Guterstam et al., 2015; Park and Blanke, 2019), and insula (Bottini and Salvato, 2020; Clemente et al., 2014; Corradi-Dell’Acqua et al., 2008; Ehrsson, 2007; Guterstam et al., 2015; Serino et al., 2013). Fourth, within this mediating network of brain areas, conditions known to affect embodiment should affect brain areas and downstream networks associated with presence, but not necessarily vice versa. Manipulations of presence were not necessarily expected to affect brain regions associated with embodiment.

Regions of interest (ROI) for these brain areas were derived from the standard MNI atlas. The high-resolution ROIs from the anatomical reference volume were resampled to match the resolution of the EPI data. For each ROI, the model parameters corresponding to the estimates of mean percent change in BOLD signal (beta weights) were computed for each condition (e.g., HE) and each run. Ten runs were excluded based on outliers that were more than three standard deviations from the mean (nearly all these outliers were several orders of magnitude larger than expected, and possibly reflect artifacts). The beta weights for the remaining runs (*N* = 34) were used in subsequent analyses. Mean beta weights for each condition were computed, and *a priori post hoc* comparisons (Bonferroni corrected *t*-tests, a_CRIT_ = 0.025) were made between relevant conditions: HE vs. LE; HP vs. LP; and HE vs. HP (control).

## 3 Results

### 3.1 Psychophysical experiments

**FIGURE 2:**
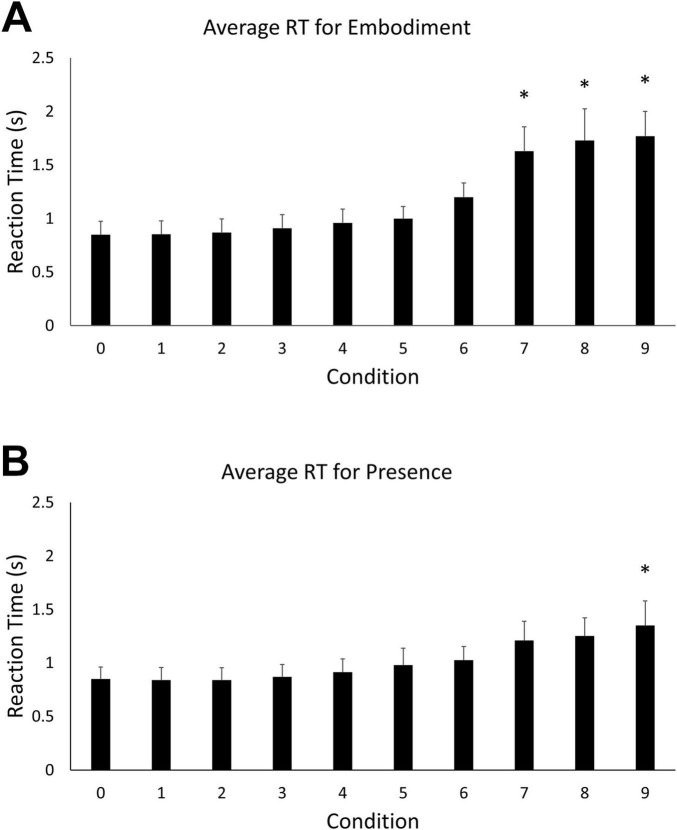
**(A)** Mean reaction time for the embodiment condition. Error bars indicate 95% confidence intervals around the mean. Asterisks indicate averages that were significantly different from step 0. **(B)** Mean reaction time for the presence condition. Asterisks indicate averages that were significantly different from step 0.

Mean reaction time and accuracy were computed for each of the ten levels of the two main BSC conditions (embodiment vs. presence). An omnibus one-way ANOVA revealed a statistically significant difference in reaction times among the ten steps of the embodiment condition, *F* (9, 100) = 18.303, *p* < 0.05. Tukey’s HSD Test for multiple comparisons found that the mean reaction time for embodiment step 0 (*M* = 0.85 s, *SD* = 0.21) was significantly different than when the motion vector was rotated 70 degrees (*M* = 1.63 s, *SD* = 0.38) [*q* (9, 100) = –0.78, *p* < 0.05], 80° (*M* = 1.73 s, *SD* = 0.5) [*q* (9, 100) = –0.88, *p* < 0.05], and 90° (*M* = 1.77 s, *SD* = 0.39) [*q* (9, 100) = –0.92, *p* < 0.05] (Figure 2A). These behavioral data indicate that performance decreases with increasing error in the visual feedback signal for real arm movements. A similar omnibus one-way ANOVA revealed a statistically significant difference in reaction times among the ten steps of the presence condition, *F* (9, 100) = 3.651, *p* < 0.05. Tukey’s HSD Test for multiple comparisons found that the mean value of step 0 (*M* = 0.85, *SD* = 0.19 s) was significantly different from step 9 (*M* = 1.769, *SD* = 1.45 s), *q* (9, 100) = –0.92, *p* < 0.05) (Figure 2B). An outlier was observed for presence step 9 (> 3 standard deviations from the mean). The decision to evaluate outliers was based on visual inspection of the data. For every step condition, data that were greater than three standard deviations from the mean were considered for removal. However, the observed outlier was the only data point that exceeded the criterion. This data point was removed and the comparisons between steps 0 and 9 were reanalyzed. A significant difference was still observed between the mean value for step 0 (*M* = 0.85, *SD* = 0.19 s) and step 9 (*M* = 1.35, *SD* = 0.39 s), *p* < 0.05.

**FIGURE 3:**
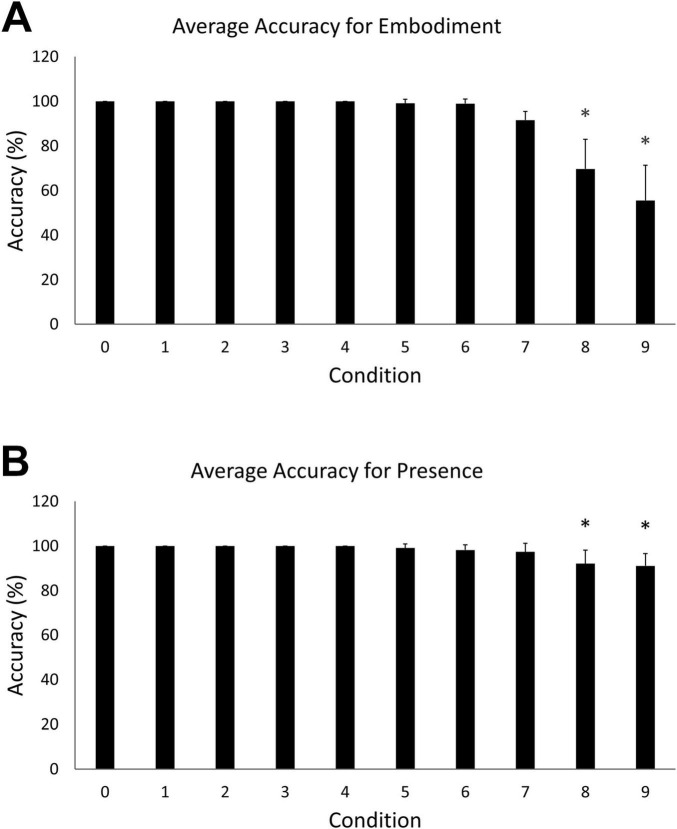
**(A)** Mean accuracy for the embodiment condition. Error bars indicate 95% confidence intervals around the mean. Asterisks indicate averages that were significantly different from step 0. **(B)** Mean accuracy for the presence condition. Asterisks indicate averages that were significantly different from step 0.

A one-way ANOVA revealed a statistically significant difference in embodiment accuracy scores among the ten steps, *F* (9, 100) = 21.23, *p* < 0.05). Tukey’s HSD Test for multiple comparisons found that the mean accuracy for embodiment step 0 (*M* = 100, *SD* = 0) was significantly different than embodiment steps 8 (*M* = 69.7, *SD* = 22.38) [*q* (9, 100) = 30.3, *p* < 0.05] and 9 (*M* = 55.85, *SD* = 26.7) [*q* (9, 100) = 44.1, *p* < 0.05] (Figure 3A). A statistically significant difference in accuracy scores was also observed among the ten steps of the presence condition, *F* (9, 100) = 4.97, *p* < 0.05. Tukey’s HSD Test for multiple comparisons found that the mean accuracy for step 0 (*M* = 100, *SD* = 0) was significantly different than steps 8 (*M* = 92.1, *SD* = 10.3) [*q* (9, 100) = 7.93, *p* < 0.05] and 9 (*M* = 91.1, *SD* = 9.24) [*q* (9, 100) = 8.93, *p* < 0.05] (Figure 3B).

### 3.2 Physiological experiments

#### 3.2.1 Behavioral data

**FIGURE 4:**
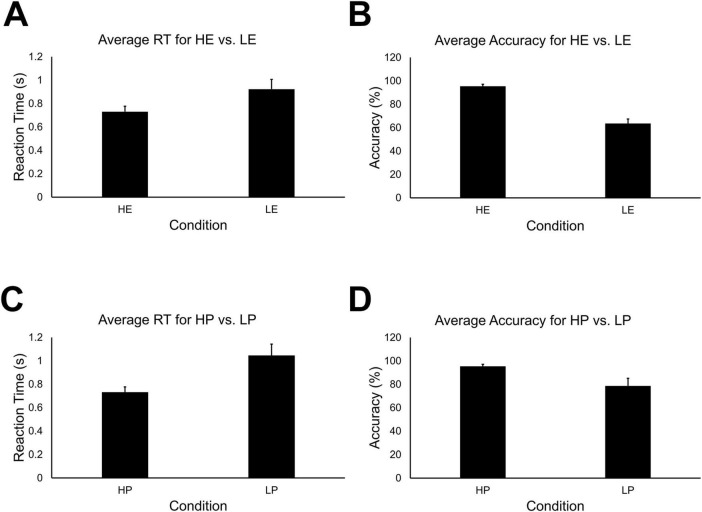
**(A)** Mean reaction time for high-embodiment and low-embodiment fMRI conditions. Error bars indicate 95% confidence about the mean. There was a significant effect for embodiment on the average reaction time, *p* < 0.05. **(B)** Mean accuracy for high and low embodiment conditions. There was a significant effect for embodiment on the average accuracy, *p* < 0.05. **(C)** Mean reaction time for high and low presence conditions. There was a significant effect for presence on the average reaction time, *p* < 0.05. **(D)** Mean accuracy for high and low presence conditions. There was a significant effect for presence on mean accuracy, *p* < 0.05.

Statistical analyses were conducted for 44 total runs across all 12 participants. An error in data collection resulted in the loss of some behavioral data for the high embodiment condition. Consequently, because stimuli for the two conditions were identical, the high presence condition was used as a surrogate for the high embodiment condition. Welch’s test revealed there were no differences between high-embodiment (*M* = 0.94; *SD* = 0.52) vs. high-presence (*M* = 0.73; *SD* = 0.16) for reaction time, *t*(df = 21) = 1.40, *p* > 0.05. And there were no differences between high-embodiment (*M* = 84.86; SD = 20.90) vs. high-presence (*M* = 95.46; *SD* = 5.88) for accuracy, *t*(df = 21) = 1.29, *p* < 0.05. A 2 × 2 repeated measures ANOVA for reaction time data did not reveal a significant main effect for embodiment vs. presence conditions, *F*(1, 172) = 2.68, *p* > 0.10. A significant main effect was observed for high- vs. low-immersion [*F*(1,172) = 46.77, *p* < 0.05], and a significant interaction effect was not observed for condition type vs. immersion level, *F*(1,172) = 2.68, *p* > 0.10. *Post hoc* pairwise comparisons revealed significant differences for high- (*M* = 0.73; *SD* = 0.16) vs. low-embodiment (*M* = 0.92; *SD* = 0.28) conditions (Figure 4A) and for high- (*M* = 0.73; *SD* = 0.16) vs. low-presence (*M* = 1.04; *SD* = 0.33) conditions (Figure 4C), all *p* < 0.05.

A 2 × 2 repeated measures ANOVA for mean accuracy revealed significant main effects for embodiment vs. presence condition types [*F*(1,172) = 13.95, *p* < 0.05] and high- vs. low-immersion levels, *F*(1,172) = 144.48, *p* < 0.05. A significant interaction effect was also observed for condition type and immersion level, *F*(1,172) = 13.95, *p* < 0.05. *Post hoc* pairwise comparisons revealed significant differences between high- (*M* = 95.42; *SD* = 5.67) vs. low-embodiment (*M* = 63.56; *SD* = 13.16) conditions (Figure 4B). Significant differences were also observed between high- (*M* = 95.42; *SD* = 5.67) vs. low-presence (*M* = 78.67; *SD* = 21.96) conditions (Figure 4D), all *p* < 0.05.

#### 3.2.2 fMRI data

**FIGURE 5:**
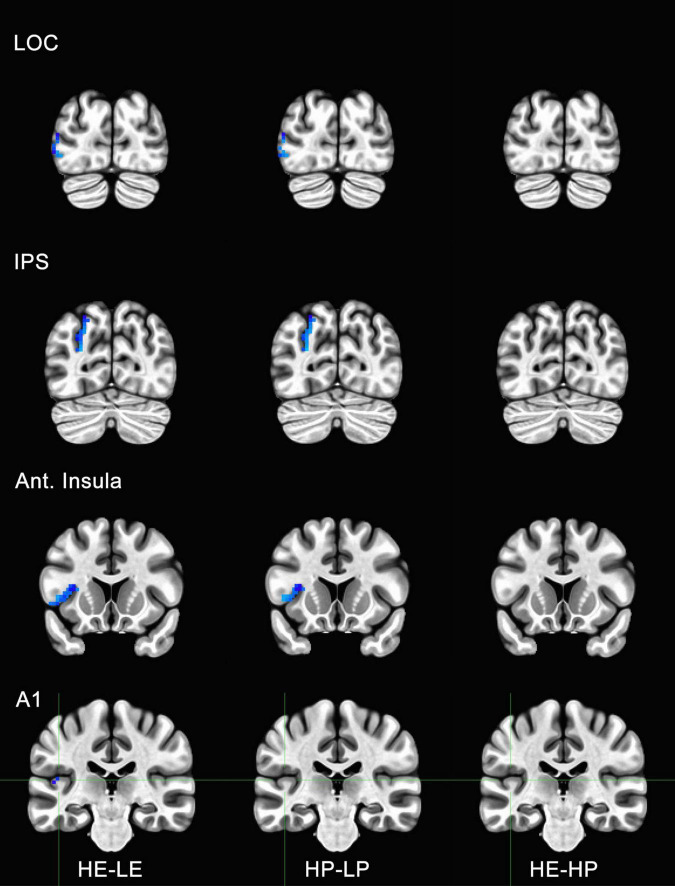
Functional magnetic resonance imaging (fMRI) responses to manipulations of presence and embodiment in lateral occipital cortex (LOC), intraparietal sulcus (IPS), insula, and primary auditory cortex (A1). Blue pixels indicate voxels that were significantly different for comparisons between high-embodiment (HE) vs. low-embodiment (LE) or high-presence (HP) vs. low-presence (LP). Critical regions (LOC, IPS, and insula) were significantly active for manipulations to both embodiment and presence. There were no significant voxels between identical conditions (HE vs. HP) or area A1, which was not predicted to show a difference between conditions.

**FIGURE 6:**
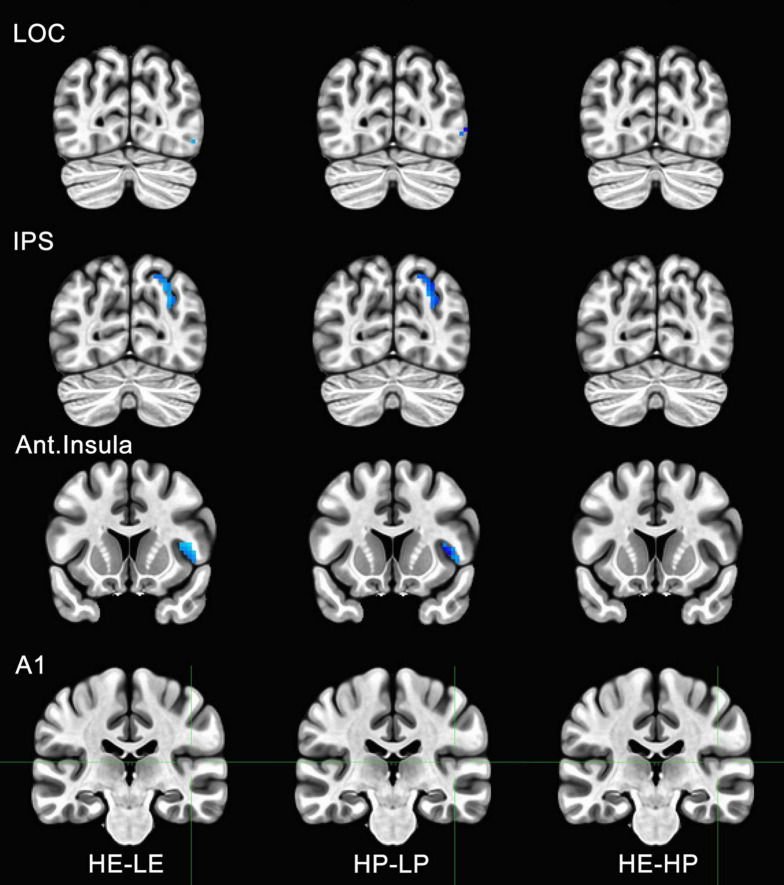
Functional magnetic resonance imaging (fMRI) responses to manipulations of presence and embodiment in lateral occipital cortex (LOC), intraparietal sulcus (IPS), insula, and primary auditory cortex (A1). Blue pixels indicate voxels that were significantly different for comparisons between high-embodiment (HE) vs. low-embodiment (LE) or high-presence (HP) vs. low-presence (LP). Critical regions (LOC, IPS, and insula) were significantly active for manipulations to both embodiment and presence. There were no significant voxels between identical conditions (HE vs. HP) or area A1, which was not predicted to show a difference between conditions.

Analysis of the group data was conducted in AFNI using a mixed-model, three-factor ANOVA. These data were also used to visualize the data (Figures 5, 6). Two fixed-effects factors and one random-effects factor were included. The first fixed-effect factor was the BSC component (presence vs. embodiment). The second fixed-effect factor was the level of immersion (high vs. low). Together, the levels of these factors map onto our four stimulus conditions: HE, LE, HP, and LP. The random-effects factor of the model corresponds to the beta weights for each run for each participant. The three-way ANOVA computes a voxel-wise omnibus F test, main effects for each factor, interaction effects, and *post hoc* contrasts for relevant comparisons: HE vs. LE; HP vs. LP; and HE vs. HP (control condition).

After preprocessing data for each run, the mean percent change in BOLD signal was computed for several regions of interest (Tables 1, 2). BOLD data acquired for each condition was compared with its complementary condition (i.e., HE vs LE and HP vs. LP) using a paired *t*-test with a Bonferroni correction for multiple comparisons (α = 0.025). While the anatomical reference volume was in standard MNI coordinates, ROIs were derived from either that atlas or the Talairach and Tournoux atlas (Lancaster et al., 2000) that comes installed with AFNI (Talairach, 1988). If ROIs were not available from either of these standard atlases, MNI coordinates from previous BSC studies were used. The names of analogous ROIs are indicated in the middle column if the atlas used a different name from the one specified in our model or the models of others. Behavioral and physiological data were also compared for each ROI across participants and runs (Supplementary materials).

**TABLE 1:** The table below shows the significant regions of interests (ROIs) observed for embodiment contrasts.

Embodiment ROIs		
Region of interest	ROI source	*P*-value (* = *p* < 0.025)
L. hippocampus	TT_Daemon	0.160
L. inferior parietal lobe	TT_Daemon	< 0.001*
L. insula	TT_Daemon	< 0.001*
R. insula	TT_Daemon	0.007*
L. intraparietal sulcus	-22, -56, 54 from Guterstam et al. (2015)	< 0.001*
R. intraparietal sulcus	32, -54, 54 from Guterstam et al. (2015)	< 0.001*
L. posterior cingulate	-9, -54, 44 from Guterstam et al. (2015)	0.071
R. posterior cingulate	6, -33, 48 from Guterstam et al. (2015)	0.174
L. lateral occipital cortex	-42, -74, -8 from Guterstam et al. (2015)	0.012*
R. lateral occipital cortex	48, -66, -12 from Guterstam et al. (2015)	< 0.001*
L. inferior precentral sulcus (PMv)	L_Ventral_Area_6from MNI Atlas	< 0.001*
L. anterior intraparietal sulcus	L_Area_7PCfrom MNI Atlas	< 0.001*
L. middle frontal gyrus	TT_Daemon	0.002*
R. superior precentral sulcus (PMd)	R_Inferior_6-8_Transitional_Area from MNI Atlas	0.013*
R. middle frontal gyrus	TT_Daemon	0.002*
L. area PFt	TT_Daemon	< 0.001*
L. anterior cingulate cortex	TT_Daemon	0.002*
R. anterior cingulate cortex	TT_Daemon	0.019*
L. premotor cortex	L_Precentral_Gyrusfrom TT_Daemon	< 0.001*
L. postcentral gyrus	TT_Daemon	< 0.001*
L. anterior insula	L_Frontal_Opercular_Area_4 from MNI Atlas	< 0.001*
R. anterior insula	R_Frontal_Opercular_Area_4 from MNI Atlas	< 0.001*
L.middle insula	L_Middle_Insular_Areafrom MNI Atlas	0.001*
R. mid dle insula	R_Middle_Insular_Areafrom MNI Atlas	< 0.001*

Asterisks indicate statistically significant contrasts (*p* < 0.025).

**TABLE 2:** The table below shows the significant regions of interests (ROIs) observed for presence contrasts.

Presence ROIs		
Region of interest	ROI source	*P*-value (* = *p* < 0.025)
R. hippocampus	TT_Daemon	0.422
L. hippocampus	TT_Daemon	0.145
L. insula	TT_Daemon	< 0.001*
R. insula	TT_Daemon	0.011*
L. intraparietal sulcus	-22, -56, 54 from Guterstam et al. (2015)	< 0.001*
R. intraparietal sulcus	32, -54, 54 from Guterstam et al. (2015)	< 0.001*
L. Posterior Cingulate	-9, -54, 44 from Guterstam et al. (2015)	0.645
R. posterior cingulate	6, -33, 48 from Guterstam et al. (2015)	0.313
L. precuneus	TT_Daemon	0.644
R. precuneus	TT_Daemon	< 0.001*
L. retrosplenial cortex	MNI Atlas	0.552
R. retrosplenial cortex	MNI Atlas	0.103
L. TPJ	MNI Atlas	0.007*
R. TPJ	-42, -74, -8 from Guterstam et al. (2015)	< 0.001*
L. lateral occipital cortex	-42, -74, -8 from Guterstam et al. (2015)	< 0.001*
R. lateral occipital cortex	48, -66, -12 from Guterstam et al. (2015)	< 0.001*
L. supramarginal gyrus	L_Inferior_Parietal_ Lobulefrom TT_Daemon	< 0.001*
R. middle frontal gyrus	TT_Daemon	< 0.001*
R. superior precentral sulcus	MNI Atlas	< 0.001*
L. parietal operculum	L Inferior Frontal Gyrusfrom TT_Daemon	0.065
L. posterior insula	L_Posterior_Insular_Area_2 from MNI	0.044
L. postcentral gyrus	TT_Daemon	< 0.001*
R. postcentral gyrus	TT_Daemon	< 0.001*
L. anterior insula	L_Frontal_Opercular_Area_4 from MNI Atlas	< 0.001*
R. anterior insula	R_Frontal_Opercular_Area_4 from MNI Atlas	< 0.001*
L. middle insula	L_Middle_Insular_Areafrom MNI Atlas	< 0.001*
R. middle insula	R_Middle_Insular_Areafrom MNI Atlas	< 0.001*

Asterisks indicate statistically significant contrasts (*p* < 0.025).

#### 3.2.3 Control experiments

To determine if the experimental manipulation was truly influencing brain regions involved with embodiment and presence, regions that were not related to these behaviors were analyzed. The principal motivation was to ensure that any effects associated with BSC were not the result of measurement artifacts or spontaneous neuronal activity. Several ROIs were considered, including visual areas not typically associated with BSC (e.g., V3, V4, MT, IT). However, there was a concern these areas might be sensitive to unforeseen differences between conditions (e.g., subtle differences in visual motion generated by the participant). Polysensory areas and areas with connections to multiple sensory systems (e.g., thalamus) were also avoided. Hence, the left and right parabelt complex and primary auditory cortex were used as ROIs and comparisons were made between the data acquired for high- and low-embodiment/presence conditions. Given that the experimental design of this study did not include audio apart from that of the scanner, which is presumed to be uncorrelated with the experimental conditions, no differences were predicted between conditions for this study. No significant activity was observed for the bilateral parabelt complex or the primary auditory cortex (e.g., Area A1 in Figures 5, 6), suggesting that the design implemented in this study only influenced the ROIs predicted to be affected.

Activity for HE and HP conditions was compared for each ROI to determine if the two conditions were truly identical. *Post-hoc* tests with Bonferroni corrections were used to determine if high embodiment and presence conditions differed from one another for the ROIs measured in this study. There were no significant differences observed for any of the ROIs measured between high embodiment and presence conditions (e.g., far right column, Figures 5, 6). Therefore, it can be assumed that there were no differences between identical conditions (HE vs. HP), which suggests that the physiological differences observed between planned comparisons (e.g., HE vs. LE) were unlikely the result of noise or chance.

#### 3.2.4 Model assessment

We proposed a coupled model for BSC, where the perception of presence is predicated upon the perception of embodiment. To assess the accuracy of this model, beta weights for ROIs associated with embodiment and presence were compared. 3dREMLfit was used to compute beta weights for each condition (i.e., HE, LE, HP, and LP). Beta weights were computed for each ROI across all runs and participants. The mean beta weights were computed for each condition, and the pooled standard deviation was computed for relevant comparisons (e.g., HE vs. LE). The effect size for relevant comparisons was computed as the absolute value of the difference between complimentary conditions divided by the pooled standard deviation. Beta weights for any given run that exceeded two standard deviations from the mean for that condition were excluded from the analysis. Many of these omissions were several orders of magnitude from the mean and likely reflected artifacts. We compared the effect of our manipulations (e.g., HE vs. LE) on the effect sizes for ROIs associated with either embodiment or presence. For all ROIs, the effect of the embodiment manipulation on presence ROIs (*M* = 0.0287; *SD* = 0.0198) was larger than that of the presence manipulation on embodiment ROIs (*M* = 0.012; *SD* = 0.0081), *t*(50) = 3.86, *p* <0.001. The same analysis was conducted after excluding integrative ROIs (brain areas known to respond to manipulations of both embodiment and presence). Very few ROIs remained after this elimination (*n* = 7 for embodiment ROIs and *n* = 11 for presence ROIs). Nevertheless, the *t*-test is robust when used with small sample sizes, and bootstrapping is not required (de Winter, 2013). Levine’s test revealed unequal variance between groups, *F*(16) = 5.931, *p* = 0.027. Consequently, Welch’s *t*-test, which assumes unequal variance between groups, was used. The effect size of the embodiment manipulation on presence ROIs (*M* = 0.0231; *SD* = 0.022) was larger than that for the presence manipulation on embodiment ROIs (*M* = 0.0067; *SD* = 0.0043), *t*(11.16) = 2.41, *p* = 0.034. Cohen’s d was computed (*d* = 0.94) using the pooled standard deviation (*SD* = 0.0176), which is a large effect size for the difference in mean effect sizes for each group. Thus, the effect of embodiment manipulations on ROIs associated with presence is larger than the effect of presence manipulations on ROIs associated with embodiment. Manipulations to embodiment affect presence, but not necessarily vice versa. This data supports a hierarchical model where the perception of presence is predicated upon the perception of embodiment.

The observed effect size is quite large, which is remarkable given the relatively low number of participants (Vul et al., 2009; Button et al., 2013). An analysis was conducted to determine if any data had enough leverage to produce a false positive. Cross-validation is a method for assessing the leverage of a single run, a single subject, or a portion of the data (Esterman et al., 2010; James et al., 2013). The “leave one out” approach of a cross-validation analysis removes a portion of the data and repeats the analysis to determine its influence. There are many ways to exclude data in cross-validation, but we elected to remove individual participants. This classic “leave-one-subject-out” (LOSO) approach assumes that individual subjects are more likely to exert undue influence over the data. First, the beta weights for the excluded participant were removed from all runs for every ROI. Second, the effect size of each contrast (e.g., HP vs. LP) was recomputed for each ROI using the remaining participants. Third, the effect sizes for ROIs associated with presence and embodiment were aggregated. Fourth, the integrative ROIs that were associated with both embodiment and presence were removed. Finally, we compared the effect sizes for embodiment manipulations (i.e., HE vs. LE) on presence ROIs versus presence manipulations (i.e., HP vs. LP) on embodiment ROIs. Welch’s test for the asymmetry in effect sizes was robust for all permutations of the LOSO analysis, all *p* < 0.05. Cohen’s *d* indicated the effect sizes associated with the asymmetry were large for each permutation [*Range* = (0.87, 1.08); *M* = 1.00; *SD* = 0.068]. Thus, no individual participant had undue leverage over our assessment of the hierarchical model of BSC. Moreover, because the effect size was relatively consistent for all LOSO permutations, we have more confidence that our conclusions are not based on spurious results.

## 4 Discussion

### 4.1 Psychophysical experiments

The psychophysical experiments used an HMD to demonstrate that manipulations of sensory feedback and perspective profoundly affect performance measures associated with perceptions of BSC in a virtual world. Relative to first-person viewing conditions with correlated feedback, performance was negatively affected for embodiment conditions with uncorrelated sensorimotor feedback and for presence conditions with a third-person perspective. These findings are consistent with other studies that have explored aspects of embodiment with the use of correlated and uncorrelated multisensory feedback. For example, Ratcliffe and Newport (2017) reported that limb ownership was greater during synchronous correlated feedback compared to uncorrelated feedback conditions. This observation is also consistent with studies that used the rubber hand illusion to measure embodiment (Armel and Ramachandran, 2003; Botvinick and Cohen, 1998; Costantini and Haggard, 2007; Lloyd, 2007). When a fake or virtual arm is presented at a plausible location in space, depending on the degree of multisensory conflict visualized by the person, participants may embody the fake/virtual arm. Kilteni et al. (2012) also found participants could even feel ownership of a virtual arm that was three times longer than their actual arm.

The results of our psychophysical experiments are also consistent with studies that manipulated presence by altering perspective. Reports of presence in these studies were higher when viewing from a first- versus a third-person perspective (Ionta et al., 2011; Lenggenhager et al., 2007; Maselli and Slater, 2013; Slater et al., 2010). The perceptual conflict created between visual and tactile stimulation created the illusion that participants were no longer present at their physical location, but instead felt situated at the location of the image presented in the HMD (Lenggenhager et al., 2007). This illusion might arise because BSC is dynamic and can alter depending on changes to various sensory signals.

There may have been some issues with this study that limit our interpretation of the results. Only eleven participants were recruited for this study because participation was limited by restrictions during the COVID-19 pandemic. Statistical power was achieved by collecting more data per participant. However, recruiting a larger sample size might have revealed greater differences. Additionally, no direct self-report of BSC was obtained, and therefore all perceptions of BSC are inferred by performance data. It is likely that the degradation in performance we observed at increased step sizes co-varied with diminished perceptions of embodiment and presence. The presumptive correlation between performance and subjective reports is based on previous studies in the literature where subjective reports were obtained along with performance measures on a motor task similar to ours (Ishikawa et al., 2021).

### 4.2 Physiological experiments

#### 4.2.1 Behavioral data

The psychophysical data from our physiological experiments were consistent with the findings observed in our preliminary psychophysical experiments. Performance was relatively worse for low-immersion conditions that were designed to disrupt embodiment or presence. Consistent with previous studies that have altered embodiment, manipulating the sensorimotor feedback between the real and illusory arm destroys the embodiment illusion and results in slower reaction times and relatively poor accuracy (Kilteni et al., 2012). Similarly, changing from first- to third-person perspective negatively affected performance in presence conditions, which has been reported in the literature as well (Ehrsson, 2007; Ionta et al., 2011; Maselli, 2015). The behavioral results from our physiological experiments are not only notable because they are similar to the behavioral results of other BSC studies, but also because this paradigm is specific to virtual worlds. These findings suggest that cues supporting multisensory integration are similar for real and virtual worlds.

#### 4.2.2 Physiological data

Comparisons between high-embodiment and low-embodiment (HE vs. LE), and high-presence and low-presence (HP vs. LP) conditions provide evidence that the perception of embodiment and presence derive from shared, overlapping neural networks (Tables 1, 2). Significant changes in BOLD signal activation in response to alterations in high- and low-embodiment conditions were observed within the left inferior parietal lobule, left inferior precentral sulcus (PMv), anterior intraparietal sulcus, middle frontal gyrus, right superior precentral sulcus (PMd), left PFt, anterior cingulate cortex, left premotor cortex, and the left postcentral gyrus. Significant neurophysiological changes were also observed in response to differences between high- and low-presence conditions within the right precuneus, temporoparietal junction, left supramarginal gyrus, right middle frontal gyrus, right superior precentral sulcus, and the postcentral gyrus. As predicted by our proposed model, changes in the BOLD signal co-varied with changes to both presence and embodiment conditions within the insula, intraparietal sulcus, and lateral occipital cortex. However, no changes were observed in the posterior cingulate cortex.

#### 4.2.3 Coupled network models

Coupled network models assert that perceptions of embodiment and presence derive from overlapping, shared brain regions. Consistent with Guterstam et al. (2015), our physiological experiments demonstrate that intraparietal sulcus, lateral occipital cortex, left inferior precentral sulcus (PMv), left anterior intraparietal sulcus, and right superior precentral sulcus (PMd) contribute to the experience of embodiment. Also consistent with Guterstam et al. (2015) our data suggest that right intraparietal sulcus, left supramarginal gyrus, right middle frontal gyrus, and right superior precentral sulcus contribute to the perception of presence. However, we did not find evidence that hippocampus, posterior cingulate cortex, or left precuneus supports the perception of presence. Activity in the IPS was also correlated with both embodiment and presence conditions. However, unlike Guterstam et al. (2015), significant activation was observed for both embodiment and presence within the insula and LOC, and the PCC was not found to be significantly active.

Park and Blanke (2019) proposed a coupled network model that differed from Guterstam et al. (2015) in that the insula, rather than the PCC, was implicated in the perceptions of both embodiment and presence. Though, both models suggest that the IPS is an important contributor to the perception of embodiment and presence. Similar to Park and Blanke (2019), the insula and intraparietal sulcus showed significant changes in BOLD activation during changes to both embodiment and presence conditions. Additionally, activity in temporal parietal cortex only corresponded to changes in presence conditions. However, unlike Park and Blanke (2019), we did not find significant changes in posterior cingulate cortex for either embodiment or presence conditions.

Blanke et al.’s (2015) model for BSC posits that the insula and anterior cingulate cortex are also associated with the perception of embodiment, and the temporoparietal junction correlates with the perception of presence. The anterior cingulate cortex was not reported in Park and Blanke’s (2019) model. Regions of interest that were significantly different between embodiment conditions that support Blanke et al. (2015) include the premotor cortex, insula, intraparietal sulcus, and anterior cingulate cortex. Areas consistent for presence in this study include the temporoparietal junction, and the supramarginal gyrus. However, we observed an overlap between perceptions of embodiment and presence within the intraparietal sulcus, lateral occipital cortex, and insula that was not reported in Blanke et al. (2015).

#### 4.2.4 Decoupled networks models

Decoupled network models propose that embodiment and presence culminate from separate neural networks with no specific cite of integration. The decoupled model proposed by Serino et al. (2013) is derived primarily from the data from Ionta et al. (2011) and Petkova et al. (2011a) and asserts that body ownership is encoded in premotor cortex and body location is represented in temporoparietal junction. Like Serino et al. (2013), we report significant activity in brain regions associated with the perception of embodiment, including the inferior parietal lobe, lateral occipital cortex, postcentral gyrus, ventral premotor cortex, and the insula. Significant BOLD signal activation was seen in brain areas known to support presence, specifically the temporoparietal junction. However, unlike Serino et al. (2013) and consistent with coupled models of BSC, activation within insula, lateral occipital cortex, and the left postcentral gyrus appeared to support the perceptions of both presence and embodiment. This pattern suggests that regions of overlap mediate the flow of information between brain regions associated with embodiment and presence.

#### 4.2.5 Cumulative evidence for a coupled network model

The results from our physiological experiments suggest that brain networks supporting the perception of embodiment and presence are coupled. When presented with plausible yet incongruent sensorimotor feedback, bottom-up signals that are integrated by multimodal brain regions — including the premotor cortex, insula, PCC, IPS and LOC — govern the perception of embodiment. When the visual perspective of a real or virtual body is manipulated (e.g., third- vs. first-person viewpoint), neuronal activity in the temporoparietal junction, precuneus, insula, and lateral occipital cortex are altered to affect perceived presence. Finally, we provide some evidence that the intraparietal sulcus, insula, and lateral occipital cortex mediate perceptual experiences of both embodiment and presence. It is our belief that the interaction between these networks culminates into a unitary experience of BSC. With a few exceptions, these findings are consistent with our proposed model of BSC described below.

It is also possible that task-specific differences might explain discrepancies between this study and those that support decoupled models of BSC. There are relatively few that champion fully decoupled models of BSC. Curiously, the evidence supporting decoupled models of BSC is often accompanied by interactions between embodiment and presence under certain circumstances. We argue that these observations are not irregularities but, perhaps, indications of a relationship between the two systems. We address three such models by Longo et al. (2008), Serino et al. (2013), and Maselli and Slater (2014). Longo et al. (2008) took a psychometric approach to the rubber hand illusion. Principal components analysis (PCA) of the participants’ subjective reports indicated the illusion could be attributed to three factors: ownership, agency, and location. Location and ownership could independently predict the bias in the illusion. The authors concluded that there was a dissociation between limb ownership and self-location. However, the authors also reported that the two perceptions were typically correlated, and the dissociation between the ownership and location occurred at later stages of processing. Notably, the factor of ownership also predicted the degree of drift in self-location reports, which suggests that self-location may partially depend upon embodiment after all. This evidence appears to contradict a fully decoupled model of BSC.

Serino et al. (2013) proposed a decoupled model based on the data of Ionta et al. (2011) and Petkova et al. (2011a). A double dissociation between self-location and limb ownership was observed between the two studies. However, manipulations of embodiment and presence were not conducted in the same individuals. Therefore, the evidence supporting a fully decoupled model of BSC is limited. Ionta et al. (2011) demonstrated that illusory changes in self-location induced with an out-of-body (OBE) illusion were correlated with activity in the TPJ. Bilateral TPJ was the only brain area with activity that uniquely correlated with self-location. The right extrastriate body area (EBA) was the only region uniquely associated with self-identification. This data was used to support a decoupled model of BSC. Our data correlating presence with the TPJ are similar to those of Ionta et al. (2011). However, we report increases in EBA activity associated with embodiment. They admit that the decreased activity in EBA associated with self-identification contradicts much of the literature. More importantly, the evidence supporting EBA as the location for self-identification is relatively weak. Specifically, their experiment manipulates perceived location via the OBE, but there is no independent manipulation of limb ownership. Additionally, the physiological investigation of self-identification was guided largely by responses to a single prompt in their questionnaire. These self-location and self-identification reports are potentially confounded and might also suffer from demand characteristics (Forster et al., 2022a). Petkova et al. (2011a) found that subjective reports of body ownership in a body-swap illusion correlate with activity in the bilateral ventral premotor cortex (PMv). The “body swap” illusion is a full-body ownership illusion (FBOI) where the participant views tactile stimulation of a mannequin from the mannequin’s point of view while receiving similar tactile stimulation to their own body. Synchronous visuotactile stimulation results in the perception that part, or all, of the mannequin’s body belongs to the participant. The strength of the illusion was significantly correlated with BOLD responses in bilateral PMv. On its own, the data from Petkova et al. (2011a) identifies brain regions associated with embodiment. However, there was no investigation into the neural correlates of presence. Consequently, this study alone cannot provide evidence for a decoupled model of BSC.

Maselli and Slater (2014) manipulated embodiment and presence in the same individuals in a FBOI with different perspectives: first-person, third-person, and overlapping. In the overlapping condition, the real and virtual bodies partially overlapped. Body ownership and self-location were independently altered, and embodiment and presence appeared to be encoded by different neural networks. However, manipulations to embodiment also affected presence. Despite any task differences between our study and theirs, the interaction between embodiment and presence seems to support partially coupled rather than fully decoupled models of BSC. In the partially overlapping viewing condition, simultaneous manipulations of embodiment and presence could be induced. Participants experienced a strong sense of body ownership and location at the avatar in this viewing condition, and the location effect was stronger than in the non-overlapping third-person condition. The authors concluded that, in such cases, the cues for embodiment can drive the cues for presence. This conclusion appears to contradict a fully decoupled model of BSC. Nevertheless, the authors did not record physiological data. They speculated that embodiment is encoded by visuo-proprioceptive neurons in PMv, EBA, PPC, insula, and S1, and self-location is encoded by visuotactile neurons in TPJ. While the authors demonstrated that embodiment and presence can be independently manipulated, and that they appear to be encoded by different neuronal populations in different brain areas, the authors do not rule out the possibility that these systems may be coupled.

The studies here that support decoupled models of BSC either didn’t manipulate embodiment and presence in the same participants, didn’t record physiological data, or they found some evidence of an interaction between embodiment and presence. We believe that arguments for fully decoupled models were made early in the study of BSC, when operational definitions for components of BSC were changing and investigators may have placed too much emphasis on dissociating the two constructs. Evidence suggests different neural networks support the various components of embodiment and presence, and we need to determine exactly how these networks interact. Embodiment and presence might be processed independently, both before and after a point of integration (a “bottleneck”). While this independent processing may occur before a final body representation is formed, it seems that experiencing presence depends, at least in part, on having a sense of embodiment.

#### 4.2.6 Potential sites of integration

The hallmark of coupled network models is that a network of brain areas serves to mediate the flow of activity between areas that encode embodiment and presence. In the coupled model proposed here, we propose that information flows mainly in one direction. Specifically, because it was postulated that the perception of presence is predicated upon the perception of embodiment, the following areas were predicted to govern the flow of neural activity from regions associated with embodiment to those associated with presence.

##### 4.2.6.1 Insula

We reported that the middle and anterior insula were correlated with changes to both embodiment and presence conditions. Several studies have found that the insula may account for a variety of experiences related to BSC (Aspell et al., 2013; Bernasconi et al., 2018; Clemente et al., 2014; Corradi-Dell’Acqua et al., 2008; Ehrsson et al., 2007; Ionta et al., 2011, 2014; Park and Blanke, 2019; Ronchi et al., 2015; Serino et al., 2013; Tsakiris et al., 2007). The bimodal and trimodal neurons within insular cortex may play a pivotal role in the integration of multiple sensory modalities, including vestibulation, touch, and vision (Ehrsson et al., 2007; Grivaz et al., 2017). The effectiveness of embodiment illusions is correlated with activity in the left insula (Ehrsson et al., 2007). Activity in the insula also changes when the sensory correlated feedback between the real and fake/virtual hand varies (Corradi-Dell’Acqua et al., 2008). Heartbeat awareness, and perceived changes to this experience, also correlates with activity in the insula (Ronchi et al., 2015; Tsakiris, 2017). Damage to the insular cortex has also been attributed to delusions of embodiment, such as somatophrenia, as well as autoscopic delusions and outer body experiences (Harricharan et al., 2017; Heydrich and Blanke, 2013; Wawrzyniak et al., 2018). Body disturbances that correspond to multimodal and vestibular integration, such as heautoscopy, may also coincide with insular damage. A person with heautoscopy may feel as if they see their body at a distance, and vestibular stimulation may improve body disorder symptoms (Bottini and Salvato, 2020). Taken together, and based on results from the current study, the insula may play a large role in the manifestation of BSC. It is possible that multimodal neurons in the insular cortex may communicate to frontoparietal networks contributing to embodiment, which subsequently affect the temporoparietal junction and other brain areas contributing to presence. These multimodal neurons may operate by (1) integrating vision, touch, proprioception, and vestibulation, and (2) using Bayesian statistics to update our reference of self in real space (Schwabe and Blanke, 2008).

##### 4.2.6.2 Intraparietal sulcus

Our physiological data indicate activity in the left and right intraparietal sulcus correlates with disturbances to embodiment and presence. The intraparietal sulcus contains multisensory neurons that are activated more when a stimulus approaches the hand, which may cause conflict in regions that integrate proprioceptive and visual information (Brozzoli et al., 2011; Makin et al., 2007). Neuroimaging studies have shown that prolonged synchronous visuo-tactile stimulation of a real and fake hand activated the IPS (Ehrsson et al., 2004, 2005). The results from Guterstam et al. (2015) also revealed significant decoding of self-location in the right intraparietal sulcus. In addition to hand and limb perception, it is important to note that the intraparietal sulcus has neurons that represent the face. Bremmer et al. (2001) saw intraparietal sulcus activity when visual or auditory stimuli approached the face. Petkova et al. (2011a) also reported significant changes in the intraparietal sulcus when visuo-tactile stimulation was applied to specific body parts.

##### 4.2.6.3 Posterior cingulate cortex

Our physiological experiments are not consistent with studies that associate posterior cingulate cortex with BSC. There were no significant BOLD signal changes observed in posterior cingulate cortex for embodiment or presence conditions. Guterstam et al. (2015) reported changes in posterior cingulate cortex activity during synchronous and asynchronous stroking conditions, which was taken to account for alterations in perceived embodiment. Blanke et al. (2015) reported that activity in the posterior cingulate cortex was significantly correlated with self-location, presence, and perceived head direction. Although, they argued that neural regions for embodiment are located within the PCC-IPS network, and areas for presence are in more lateral temporo-parietal regions. Their data indicate posterior cingulate cortex significantly contributed to both components of BSC. They further asserted that the posterior cingulate cortex orchestrates the flow of this between the IPS and the hippocampus. Others have speculated that the posterior cingulate cortex is a central region in the “default mode network,” and activity in this structure has been associated with decision-making, attention, memory, and other BSC related functions (Leech and Sharp, 2014). The posterior cingulate cortex may also mediate the translation between egocentric and allocentric spatial representations (Burgess, 2008). The posterior cingulate cortex and the retrosplinial cortex may work with the IPS and the hippocampus to represent the perceived spatial location of the bodily self.

Nevertheless, we did not find evidence to support these claims. One possible explanation is that our ROI for posterior cingulate cortex may not have been ideal. The ROI position was derived from coordinates of Guterstam et al. (2015) rather than a standard atlas. Additionally, the volume of the ROIs was determined using standard parameters from AFNI. If this volume was poorly positioned or the wrong size, there may have been too much noise or insufficient power to detect an effect. That said, the lateral occipital cortex and the intraparietal sulcus were analyzed using the same methods, and both areas yielded significant results.

##### 4.2.6.4 Lateral occipital cortex (extrastriate body area)

Significant changes in BOLD response in the lateral occipital cortex (EBA) were observed for our embodiment and presence manipulations. These results agree with other studies that have found activity in lateral occipital cortex relates to perception of BSC and peripersonal space. For example, the act of viewing different body parts elicited activation in areas of the EBA (Downing et al., 2001). Makin et al. (2007) also showed lateral occipital cortex activation when a visual stimulus was approaching or near the hand. Guterstam et al. (2015) revealed synchronous visuo-tactile stimulation significantly altered activity in lateral occipital cortex (EBA). Synchronous visuo-tactile stimulation to a fake hand also elicited significant changes in the lateral occipital cortex (Gentile et al., 2013). Taken together, it is possible that the lateral occipital cortex is involved in encoding multisensory information near specific body parts that intrude or interfere with the boundaries of peripersonal space.

#### 4.2.7 The hierarchical nature of BSC

Though embodiment and presence are typically defined as independent psychological constructs, our data indicate that these perceptions are supported by overlapping neural networks. The perception of embodiment is supported by frontoparietal networks that include the left inferior parietal lobule, left inferior precentral sulcus (PMv), left middle frontal gyrus, right superior precentral sulcus (PMd), left area PFt, anterior cingulate cortex, left premotor cortex, and left postcentral gyrus. Whereas the perception of presence is supported by temporoparietal networks that include precuneus, temporoparietal cortex, left supramarginal gyrus, right middle frontal gyrus, right superior precentral sulcus, and postcentral gyrus. Critically, activity in the insula, intraparietal cortex, and the lateral occipital cortex corresponded to manipulations of both presence and embodiment. Consequently, we propose that networks supporting embodiment and presence partially overlap.

**FIGURE 7:**
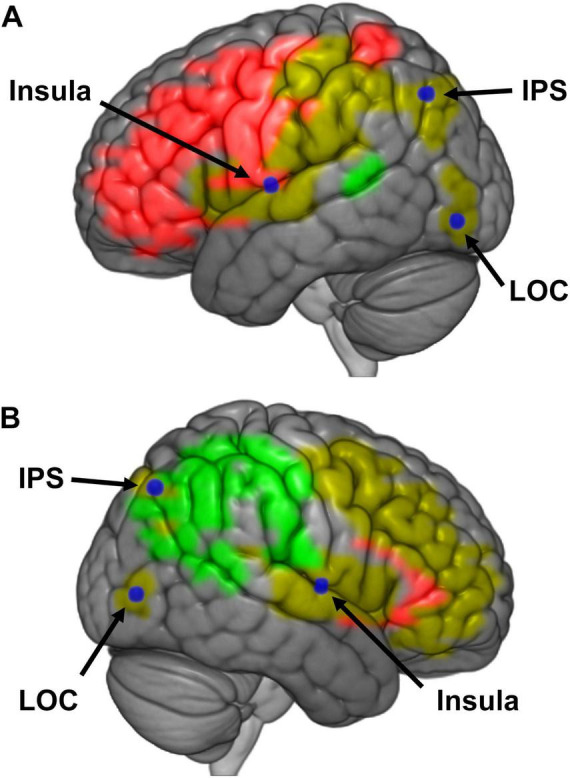
**(A)** Schematic representation of a model for bodily self-consciousness (BSC) in the left hemisphere. Regions of interests (ROIs) that were responded to manipulations of embodiment (red), presence (green), or both (yellow) are presented in color. Loci believed to mediate activity between these embodiment and presence regions are labeled in blue [i.e., insula, intraparietal sulcus (IPS), and lateral occipital cortex (LOC)]. **(B)** Schematic representation of a model for BSC in the right hemisphere.

The results also suggest that embodiment and presence are hierarchically organized, with the perception of presence depending upon embodiment. Specifically, the effect size of embodiment manipulations on brain regions associated with presence was almost four times as large as that for presence manipulations on embodiment regions. We believe that the intraparietal sulcus, insula, and lateral occipital cortex govern the flow of information between embodiment and presence regions (Figure 7). Contrary to other reports, we did not find sufficient evidence to suggest posterior parietal cortex or anterior precuneus play a role in both perceptions (Guterstam et al., 2015; Lyu et al., 2023). Rather than embodiment and presence having discrete networks that both feed into the perception of BSC, or separate networks that feed into two separate manifestations of BSC, or networks pertaining to embodiment and presence sharing overlapping neural networks via integration areas that then feed into the perception of BSC, we propose a hierarchical model of BSC – where the perception of presence is predicated upon the perception of embodiment.

It might be of value to consider an alternate hypothesis, specifically, that “one cannot perceive to be something unless they perceive to be somewhere.” If this alternative hypothesis were true, we might expect to find the following: *First, one could feel present in an environment without feeling embodied.* We speculate it would not be easy to feel present without being embodied in some form, possessing either limbs, agency, or viewpoint (the three components of embodiment according to Kilteni et al., 2012). In the absence of specific cues for presence (e.g., an empty void in a virtual world), the sense of embodiment is typically not affected. One cannot be easily severed from the multisensory cues that contribute to embodiment. In the absence of all other cues, interoceptive cues may be enough to sustain embodiment (see Park and Blanke, 2019). Forster et al. (2022b) also proposes that embodiment is a prerequisite for presence and, in cases where presence is perceived without strong cues for embodiment, a basal form of embodiment must still exist (i.e., a viewpoint). Employing the embodiment framework of Kilteni et al. (2012), the perception of presence requires having a viewpoint, but having a viewpoint does not require being present in the world. *Second, cues for presence should be necessary to create the perception of embodiment*. According to the alternate hypothesis, the perception of embodiment is predicated upon cues for presence. In previous examples, we demonstrated cues for presence are not required for the perception of embodiment (e.g., having a sense of embodiment while located in a real or virtual Ganzfeld). Conversely, the interoceptive cues for embodiment necessarily contribute to the perception of presence because they are difficult, if not impossible, to suppress in healthy, conscious individuals. *Third, the perception of presence should be more robust to changing environmental cues than the perception of embodiment*. Contrary to the alternate hypothesis, presence appears easier to manipulate than embodiment. Modern VR gaming systems demonstrate how easy it is to feel present in an infinite number of virtual worlds. However, it’s much more difficult to convince a viewer that they are a quadruped because our perception of embodiment appears quite robust. Perhaps this disparity is because presence is easily manipulated using the distal senses, whereas it is more challenging to manipulate embodiment via the proximal senses (i.e., somatosensation, interoception, proprioception). Another reason why embodiment might be less malleable is that, even though our location in the world is constantly changing, our bodies remain relatively unaltered over time. Consequently, neuronal circuits that support embodiment receive relatively predictable information regarding limb ownership, agency, and viewpoint. On the other hand, neural circuits supporting presence are constantly receiving new information as we move through the world.

It may also be valuable to compare our results to recent models that also propose hierarchical relationships between the components of BSC. There continue to be various reports exploring the relationship between embodiment and presence. For example, strong correlations between presence and embodiment have been observed using augmented reality, where both the real and virtual bodies were visible (Rosa et al., 2019). Limb ownership and presence were also found to correlate with the verisimilitude of an avatar’s hand in virtual reality (Zhang et al., 2020). A recent neuroimaging study of the rubber hand illusion also found that embodiment depends on the visibility of the avatar, the synchrony of visuotactile stimulation, and duration of stimulation (Pamplona et al., 2024). Similar to our study, many of the brain areas associated with embodiment belonged to frontoparietal networks, but some frontoparietal areas also seemed to contribute to embodiment, notably, the fusiform body area (FBA). The authors speculate that FBA might be more involved in secondary aspects of limb ownership. Areas in the TPJ are known to support presence (Arzy et al., 2006; Blanke et al., 2002; Blanke et al., 2015; Guterstam et al., 2015; Ionta et al., 2011, 2014; Park and Blanke, 2019; Serino et al., 2013; Slater et al., 2010). However, TPJ may also assist to distinguish the self from the non-self (Pamplona et al., 2022). Recently, it has been proposed that the mechanisms driving the experience of individual body part ownership may differ from those driving full-body ownership (O’Kane et al., 2024). Results from a multi-part body-ownership illusion suggest that individual body part ownership is driven by the integration of multisensory signals, particularly visual and somatosensory signals near the specific body part being considered. In contrast, full-body ownership appears to be driven by a global binding process that integrates multisensory signals from different body parts. While our study was not able to test for a hierarchical model of embodiment, this model of embodiment resonates with our hierarchical model of BSC.

Although many studies suggest that embodiment and presence arise from separate yet overlapping neural networks, others have argued that perceptions of embodiment and presence may be better described as a single, unified construct. Forster et al. (2022b) proposed the Implied Body Framework (IBF) to explain how embodiment and presence emerge from the integration of multisensory signals within the body. Their model is consistent with, but more nuanced than, a two-stage model of BSC. Multiple “implied body” representations are derived from the integration of various sensory signals (e.g., visuotactile). The plausibility of each implied body can be evaluated in relation to others, and the validity of each representation can be tested via sensory-motor feedback. Prioritized representations determine how subsequent sensory information is processed by each implied body. A second stage of multisensory integration across implied bodies yields a sense of embodiment that, when combined with other sensory information regarding allocentric space, culminates in a sense of presence. The IBF makes several predictions, one of which is the prediction that presence depends upon embodiment, and we believe we have provided preliminary evidence for this claim. Additionally, in cases where presence might be experienced without full embodiment, the authors insist that a basal form of embodiment must occur.

#### 4.2.8 Limitations and future implications

Although this study offers new clues into the underlying nature of embodiment and presence, it is not without its shortcomings. Given the time and accessibility limitations placed upon the lab during COVID, only a limited number of subjects were recruited for this study. Therefore, low statistical power might explain why some of these results differ from similar experiments. Second, this experiment was not conducted using an HMD or dichoptic images. Nevertheless, the lack of binocular disparity cues may not have been a serious limitation to immersion because participants were not asked to scan the scene nor permitted to move their head. Indeed, the behavioral data collected in the scanner was similar to that collected outside of the scanner with HMDs. It was assumed that altering sensorimotor feedback would be enough to study BSC, which is consistent with recent reports that synchronous visuomotor feedback is paramount to BSC (Penaud et al., 2023). Third, visual and tactile information from the real world could have suppressed or destroyed the BSC illusions altogether. Attention and top-down information contribute to the effectiveness of BSC illusions. Our results may have been influenced by image cues from the real world that were not fully occluded by an HMD. However, the behavioral and physiological data indicate that our manipulations influenced BSC.

Future experiments should attempt to replicate these results using an MR-compatible HMD. A stereoscopic display should elicit better immersion. Second, more participants should be recruited to improve statistical power. Third, the study could be extended with real-time subjective reports of BSC. The literature suggests that subjective measures of BSC are limited. However, future experiments might include online queries of BSC to compare with behavioral and physiological data.

While there is evidence (e.g., Matuz-Budai et al., 2022) and arguments for using subjective reports of BSC (e.g., Overgaard et al., 2023), there are several studies that call the validity of subjective reports into question (Forster et al., 2022a,b; Slater, 2004; Usoh et al., 2000). A data-driven instrument has yet to be widely accepted. The Virtual Embodiment Questionnaire (VEQ) is such a tool (Roth and Latoschik, 2020), but it has yet to be validated by a number of research groups. We were concerned that offline reports of subjective experiences could only indirectly assess the memory of embodiment and presence (Slater, 2004). We were also concerned that subjective reports might suffer from demand characteristics (Forster et al., 2022a). There was also evidence that interrupting our experiment would disrupt immersion. We conducted several pilot studies using the VEQ, but we found that, even when prompts were placed diegetically into the scene, the flow of the experience was disrupted, and our psychophysical effects were diminished. Additionally, individual prompts in subjective questionnaires can be interpreted in a variety of ways by participants (Usoh et al., 2000). Finally, we were concerned that subjective reports would severely limit the amount of data we could collect in each fMRI run. Our block-paradigm only afforded 20 s of data collection at a time, leaving very little opportunity to collect both data and subjective reports from participants.

## 5 Conclusion

An increasing amount of research has been dedicated to understanding BSC since the most recent introduction of virtual reality technology to the marketplace. Much of the physiological research on BSC has culminated in coupled or decoupled models of BSC that treat embodiment and presence as interdependent or independent, respectively. Our data supports a hierarchical model of BSC, where the perception of presence is predicated upon the perception of embodiment. This model is based on data that indicates experimental manipulations designed to alter embodiment have a far greater impact on brain regions that encode BSC than manipulations designed to affect presence, particularly in regions that encode both embodiment and presence. These data also support claims that the neural networks supporting BSC in the real world are the same as those active in virtual worlds. The findings from this study may inform future investigations of BSC and, hopefully, inform all stakeholders with an interest in virtual reality.

## Data Availability

The datasets presented in this study can be found in online repositories. The names of the repository/repositories and accession number(s) can be found below: https://doi.org/10.18112/openneuro.ds005355.v1.0.1.
