# Supplementary material for: Evidence of a hierarchical representation in bodily self-consciousness: the neural correlates of embodiment and presence in virtual worlds
Source: Front Hum Neurosci. 2025 Apr 1;19:1468947. doi: 10.3389/fnhum.2025.1468947 (PMC11996785; doi:10.3389/fnhum.2025.1468947)
Supplement: Supplementary file 1 [file Data_Sheet_1.pdf]

Supplementary Material

1 Supplementary Data

Behavioral and physiological data were compared for each ROI across participants and runs (Supplementary Table 1). The difference in means was computed for each pair of conditions (e.g., HE minus LE) for accuracy, reaction times, and BOLD signal change. fMRI data was compared to accuracy and reaction time data using simple linear regression (SLR). There were no significant correlations observed between MRI and reaction time data for the embodiment manipulation. Significant correlations were observed between MRI and accuracy data for the embodiment manipulation in the left precuneus ($r(37) = -.36$), retrosplenial cortex ($r(37) = -.36$), postcentral gyrus ($r(36) = -.34$), and premotor cortex, $r(36) = -.32$, all $p < .05$. Significant correlations were observed between MRI and reaction time data for the presence manipulation in the left and right intraparietal sulcus ($r(37) = .45$ and $r(37) = .44$), left and right precuneus ($r(39) = .38$ and $r(37) = .47$), left and right temporoparietal junction ($r(38) = .33$ and $r(38) = .32$), the right superior precentral sulcus ($r(39) = .38$), and the right middle frontal gyrus, $r(39) = .31$, all $p < .05$. Significant correlations were observed between MRI and accuracy data for the presence manipulation in the left and right intraparietal sulcus ($r(37) = -.44$ and $r(37) = -.39$), the left precuneus ($r(39) = -.41$), the left temporoparietal junction ($r(38) = -.40$), the left anterior cingulate cortex ($r(39) = -.32$), and the left parietal operculum ($r(38) = -.41$), all $p < .05$.

The pattern of correlations between reaction times, accuracy, and MRI data indicates a speed-accuracy trade-off. Overall, the mean correlation ($r_{MEAN}(34) = .10$) between reaction time and MRI data for the embodiment manipulation was positive, $t(34) = 5.87$, $p < .0001$. The mean correlation between reaction time and MRI data for the presence manipulation ($r_{MEAN}(34) = .18$) was positive, $t(34) = 7.17$, $p < .0001$. The mean correlation ($r_{MEAN}(34) = -.17$) between accuracy and MRI data for the embodiment manipulation was negative, $t(34) = -7.80$, $p < .0001$. The mean correlation between reaction time and MRI data for the presence manipulation ($r_{MEAN}(34) = -.18$) was negative, $t(34) = -7.12$, $p < .0001$. As performance decreased in the LP condition, the BOLD signal became relatively larger, which suggests that more energy was required for the brain to process information in the low-presence condition than the high-presence condition.

2 Supplementary Table 1

Embodiment Condition			Presence Condition		
**ROI**	**RT**	**Accuracy**	**ROI**	**RT**	**Accuracy**
L Hippocampus	0.11	-0.20	L Hippocampus	-0.08	-0.13
L Inferior parietal	0.11	0.06	R Hippocampus	0.17	-0.27
L Insula	0.17	-0.02	L Insula	0.03	-0.03
R Insula	0.13	-0.04	R Insula	0.15	-0.20
L IPS	0.06	-0.04	L IPS	0.45 *	-0.44 *
R IPS	0.01	-0.26	R IPS	0.44 *	-0.39 *
L PCC	0.17	-0.26	L PCC	0.17	-0.22
R PCC	0.14	-0.23	R PCC	0.22	-0.23
L LOC	-0.06	-0.05	L Precuneus	0.38 *	0.03
R LOC	0.21	0.25	R Precuneus	0.47 *	-0.41 *
L Anterior IPS	0.11	-0.17	L Retrosplenial Cortex	0.04	-0.21
L Postcentral Gyrus	0.25	-0.34 *	R Retrosplinial Cortex	0.09	-0.02
L Anterior Insula	0.07	-0.19	L TPJ	0.33 *	-0.40 *
R Anterior Insula	-0.14	-0.06	R TPJ	0.32 *	-0.16
L Middle Insula	-0.05	-0.3	L LOC	0.08	-0.19
R Middle Insula	0.04	-0.17	R LOC	0.21	-0.26
L PMv	0.12	-0.17	L Postcentral Gyrus	0.14	-0.08
L Middle Frontal Gyrus	-0.09	-0.14	R Postcentral Gyrus	0.19	-0.14
L Area PFt	0.23	-0.15	L Anterior Insula	0.28	-0.14
L ACC	0.17	-0.31	R Anterior Insula	0.23	-0.09
R ACC	0.18	-0.21	L Middle Insula	0.16	-0.13
L Premotor	0.16	-0.32 *	R Middle Insula	0.05	-0.09
R Superior Precentral Sulcus (PMd)	0.02	-0.05	R Superior Precentral Sulcus (PMd)	0.38 *	-0.26
R Middle Frontal Gyrus	-0.09	-0.14	R Middle Frontal Gyrus	0.31 *	-0.30
			L Supramarginal Gyrus	0.23	-0.02
			L Parietal Operculum	0.18	-0.41 *
			L Posterior Insula	-0.08	0.02

Supplementary Table 1: The table above shows the significant correlations between BOLD data and reaction times or accuracy for ROIs associated with embodiment and presence. Asterisks indicate statistically significant correlation coefficients ($p < .05$).
